# Association between *Interleukin-4 Receptor α Chain* (*IL4RA*) I50V and Q551R Polymorphisms and Asthma Risk: An Update Meta-Analysis

**DOI:** 10.1371/journal.pone.0069120

**Published:** 2013-07-26

**Authors:** Wei Nie, Yuansheng Zang, Jiquan Chen, Qingyu Xiu

**Affiliations:** Department of Respiratory Disease, Shanghai Changzheng Hospital, Second Military Medical University, Shanghai, China; University Heart Center Freiburg, Germany

## Abstract

**Background:**

The associations between the *interleukin-4 receptor α chain* (*IL4RA*) I50V and Q551R polymorphisms and asthma risk remained controversial.

**Methods:**

We searched the Pubmed, Embase, Chinese National Knowledge Infrastructure (CNKI) and Wanfang databases for studies published before February 2013. The strengths of the associations were calculated using odds ratios (ORs) with 95% confidence intervals (CIs).

**Results:**

A total of 50 studies were included in this meta-analysis. There was a significant association between the *IL4RA* I50V polymorphism and asthma risk in a dominant genetic model (OR = 1.13, 95% CI 1.04–1.23, *P* = 0.005). The *IL4RA* Q551R polymorphism was associated with a significantly elevated asthma risk in a recessive genetic model (OR = 1.46, 95% CI 1.22–1.75, *P*<0.0001). Subgroup analyses found that the *IL4RA* I50V polymorphism was significantly associated with asthma risk in Asians (OR = 1.72, 95% CI 1.31–2.25, *P*<0.0001), pediatric asthma risk (OR = 1.50, 95% CI 1.13–1.99, *P* = 0.005), and atopic asthma risk (OR = 1.88, 95% CI 1.27–2.79, *P* = 0.002).

**Conclusions:**

The results of this meta-analysis suggested that the *IL4RA* I50V and Q551R polymorphisms may be risk factors for developing asthma.

## Introduction

Asthma is a complex, persistent, inflammatory disease characterized by airway hyper-responsiveness and inflammation. Asthma currently represents a major public health burden in many countries [1]. Thus an understanding of the causes of this disease is an area of intense interest. Cumulative evidence supports an important genetic role in determining asthma risk [2].

T helper-2 (Th2) cytokines, such as interleukin-4 (IL-4) and IL-13, play central roles in allergic inflammation and asthma. They exert their biological activities by binding to their respective cell surface receptors, both of which share the α chain of the IL-4 receptor (IL-4Rα) [3]. Kotsimbos et al. [4] showed that expression levels of IL-4Rα messenger RNA and protein were significantly increased in the epithelium and subepithelium of biopsy specimens from subjects with atopic asthma, compared with atopic control subjects. Additionally, IL-4Rα-deficient mice were unable to produce immunoglobulin E (IgE) and the Th2 inflammatory reaction was markedly diminished [5]. Furthermore, IL-4Rα-targeted antibodies could reduce lung inflammation, airway hyper-­responsiveness and goblet-cell hyperplasia in mouse models of asthma [6]. Therefore, these results indicated that IL-4Rα may play an important role in the pathogenesis of asthma and suggested that *IL4RA* may be a strong candidate gene for asthma susceptibility.

*IL4RA* is located on chromosome 16p12.1. Many studies have investigated the associations between the *IL4RA* polymorphisms and susceptibility to asthma [7]–[56]. Most of these studies focused on two polymorphisms: I50V (rs1805010) and Q551R (rs1801275). However, the results of these studies have been controversial and inconclusive. A single study may not have sufficient power to detect slight effects of these polymorphisms on asthma because of relatively small sample sizes; however, a meta-analysis may provide more credible evidence by systematically summarizing the existing data. In 2007, Loza and Chang conducted a meta-analysis and concluded that the *IL-4RA* Q551R polymorphism, but not the I50V polymorphism, was associated with asthma risk [57]. However, that meta-analysis only included 13 studies, and several new studies with more data have been published since 2007. We therefore conducted an up-to-date meta-analysis to re-investigate the association between *IL4RA* polymorphisms and asthma risk.

## Methods

### Publication search

A literature search of the PubMed, Embase, Chinese National Knowledge Infrastructure (CNKI), and Wanfang databases was conducted for studies published before February 2013 using combinations of the following terms: (asthma or asthmatic) and (interleukin-4 receptor α chain or IL-4Rα or IL4Rα or IL4RA) and (polymorphism or mutation or variant). All eligible articles were retrieved, and their references were checked for other relevant studies.

### Study selection

All selected studies complied with the following three criteria: (1) evaluation of the *IL4RA* I50V and Q551R polymorphisms and asthma risk; (2) using a case-control design; and (3) genotype distributions in both cases and controls available for estimating an odds ratio (OR) with a 95% confidence interval (CI). If serial studies of the same population from the same group were reported, the largest study was included.

### Data extraction

Two investigators (Nie and Chen) independently extracted data from the included studies. The following information was collected from each study: first author's name, year of publication, original country, ethnicity, age group, atopic status, sample size, and genotype number in cases and controls. We verified the accuracy of the data by comparing collection forms between investigators. If different results were generated, the full text of the article was checked.

### Qualitative assessment

The quality of included studies was assessed independently by two investigators (Nie and Chen).**Table** **S1** shows the criteria for quality appraisal. The quality scoring system was based on traditional epidemiological considerations and asthma genetic issues [58]. The criteria covered the representativeness of cases and controls, the ascertainment of cases and controls, genotyping examination, Hardy-Weinberg equilibrium (HWE), and association assessment. Scores ranged from the lowest zero to the highest thirteen.

### Statistical analysis

A meta-analysis was performed when data from at least three similar studies were available. The strengths of the associations between the *IL4RA* polymorphisms and asthma risk were measured by ORs and 95% CIs. The statistical significance of summary OR was determined using the *Z* test. OR1, OR2, and OR3 were calculated for the genotypes: 1). II vs. VV (OR1), IV vs. VV (OR2), and II vs. IV (OR3) for the I50 V polymorphism, 2). RR vs. QQ (OR1), QR vs. QQ (OR2), and RR vs. QR (OR3) for the Q551R polymorphism. These pairwise differences were used to indicate the most appropriate genetic model [59]–[63]. Once the best genetic model was identified, this model was used to collapse the three genotypes into two groups (except in the case of a co-dominant model) and to pool the results.

HWE was evaluated using the Chi-square test. *P*<0.05 was considered representative of a departure from HWE. Heterogeneity of effects across studies was assessed using the Chi-square statistic and quantified by *I^2^*, which represented the percentage of total variation across studies that was attributable to heterogeneity rather than chance (*P*<0.10 was considered representative of statistically significant heterogeneity). A fixed-effect model was used when there was no heterogeneity in the studies. Otherwise, the random-effect model was used. Subgroup analyses were performed by stratifying according to ethnicity, age group, and atopic status. The stability of the results was assessed by performing a sensitivity analysis using sequential omission of individual studies. A cumulative meta-analysis was conducted by undertaking sequential pooling, starting with the earliest studies. Funnel plots were performed to estimate the potential publication bias, with an asymmetrical plot suggesting a possible publication bias. The asymmetry was assessed using the Egger's linear regression test and *P*<0.05 was considered to represent statistically significant publication bias [64]. All statistical tests were performed using STATA 11.0 software (Stata Corporation, College Station, TX). The Bonferroni correction of critical *P* values for two genetic models was applied when performing a high number of comparisons.

## Results

### Study characteristics

**Figure 1:**
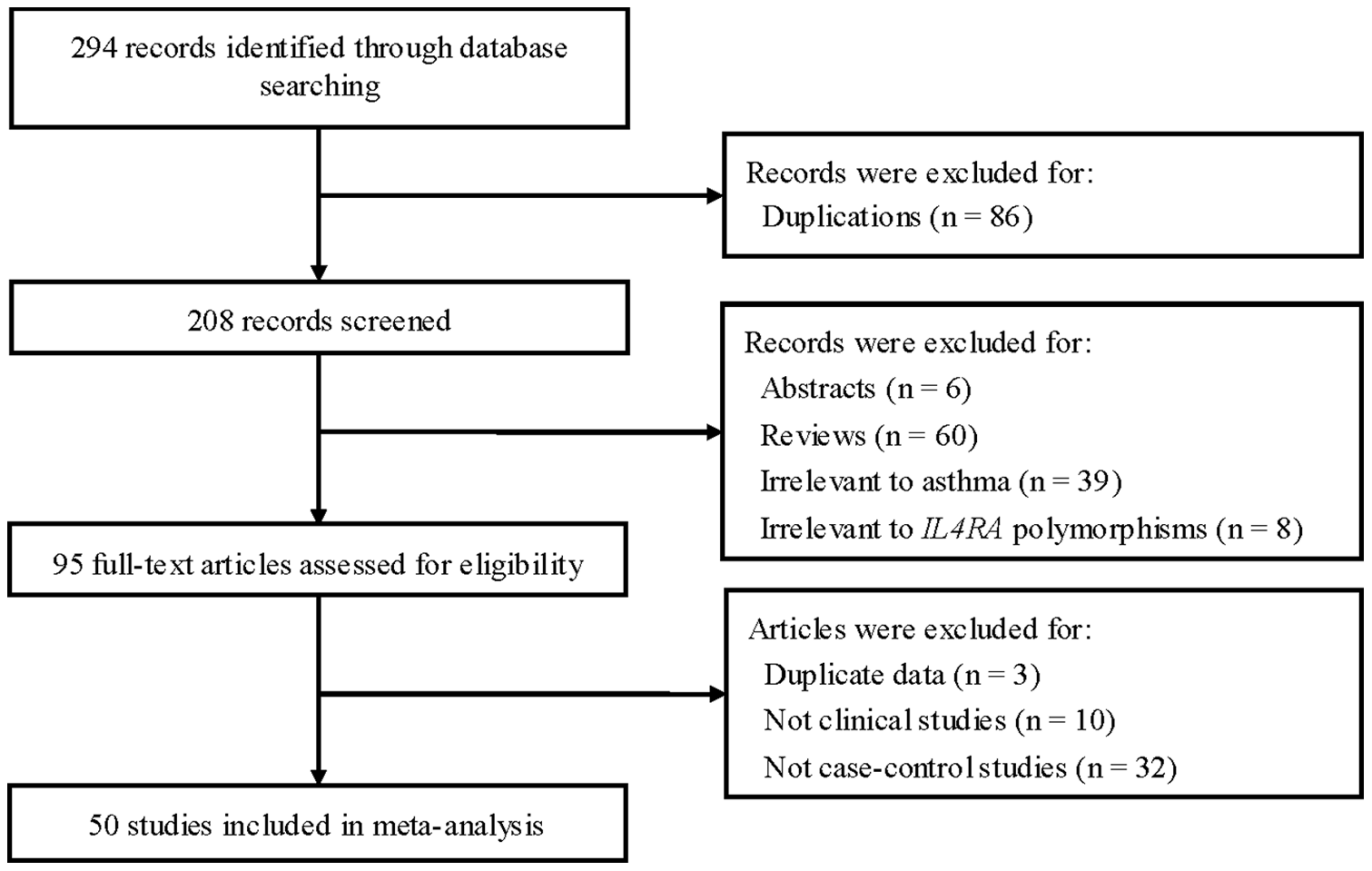
Flow of study identification, inclusion, and exclusion.

Fifty studies met the inclusion criteria [7]–[56]. A flowchart detailing the process for study identification and selection is shown in **Figure 1** **.** A study by Undarmaa et al. [51] presented two independent case-control studies, each of which was considered separately for analysis. There were 33 studies of the I50V polymorphism and 35 36 studies of the Q551R polymorphism. Twenty-seven studies were performed in Asians, 19 in Caucasians, two in Mexicans, and one in African Americans. Nineteen studies were performed in adults, and 21 in children. Twelve studies included atopic asthma patients and nine included both atopic and non-atopic asthma patients, but data for these patients could be extracted separately. The quality scores ranged from 5 to 11, suggesting that the methodological quality was generally acceptable. The characteristics of each study are presented in **Table** **1**. Genotype frequencies and HWE examination results are listed in **Table** **2**. Seven studies were not in HWE, and these studies were not included in the meta-analysis.

**Table 1:** Characteristics of the case-control studies included in meta-analysis.

First author/				Age	Atopic	Case	Control	*IL4RA*	
references	Year	Country	Ethnicity	group	status	(n)	(n)	polymorphism	Score
Mitsuyasu [7]	1998	Japan	Asian	Mixed*	Mixed*	360	120	I50V	7
Mitsuyasu [8]	1999	Japan	Asian	Mixed*	Mixed*	300	100	Q551R	7
Noguchi [9]	1999	Japan	Asian	Mixed	Atopic	101	101	I50V	9
Rosa-Rosa [10]	1999	USA	Caucasian	Adults	Mixed*	149	57	Q551R	7
Heinzmann [11]	2000	Japan	Asian	Adults	Mixed*	200	100	Q551R	8
Sandford [12]	2000	Canada	Caucasian	Adults	NA	221	143	Q551R	10
Takabayashi [13]	2000	Japan	Asian	Children	Atopic	100	100	I50V, Q551R	7
Hakonarson [14]	2001	Iceland	Caucasian	Mixed	Atopic	94	94	Q551R	10
Howard [15]	2002	Netherlands	Caucasian	Adults	NA	151	114	I50V, Q551R	9
Leung [16]	2002	China	Asian	Children	NA	76	70	I50V	9
Mújica-López [17]	2002	Mexico	Mexican	Children	Atopic	30	32	I50V	6
Risma [18]	2002	USA	Caucasian	Adults	Mixed*	200	65	I50V, Q551R	7
Beghe [19]	2003	USA	Caucasian	Mixed	NA	186	670	I50V, Q551R	7
Cui [20]	2003	China	Asian	Mixed	Atopic	241	175	Q551R	7
Hytonen [21]	2004	Sweden	Caucasian	Adults	Atopic	170	100	I50V, Q551R	6
Lee [22]	2004	Korea	Asian	Children	Mixed*	256	100	I50V, Q551R	9
Yang [23]	2004	China	Asian	Adults	NA	34	29	I50V	5
Isidoro-García [24]	2005	Spain	Caucasian	Adults	Mixed*	133	79	Q551R	8
Hu [25]	2005	China	Asian	Children	Atopic	175	175	Q551R	6
Sun [26]	2005	China	Asian	Children	NA	82	59	Q551R	6
Bernstein [27]	2006	USA	Caucasian	Adults	NA	62	75	I50V, Q551R	9
Kabesch [28]	2006	Germany	Caucasian	Children	NA	73	773	I50V	9
Melen [29]	2006	Sweden	Caucasian	Children	NA	521	509	I50V, Q551R	7
Deng [30]	2006	China	Asian	Mixed	NA	100	100	I50V	6
Gui [31]	2006	China	Asian	Children	NA	50	50	Q551R	7
Tang [32]	2006	China	Asian	Mixed	NA	103	62	I50V	5
Battle [33]	2006	USA	African American	Mixed	NA	264	176	I50V	11
López [34]	2007	Mexico	Mexican	Children	NA	88	88	I50V, Q551R	7
Mak [45]	2007	China	Asian	Adults	Mixed*	285	291	Q551R	9
Zhang W [36]	2007	China	Asian	Adults	NA	303	355	I50V, Q551R	8
Zhang H [37]	2007	China	Asian	Mixed	NA	423	114	I50V, Q551R	7
Chan [38]	2008	China	Asian	Children	NA	295	167	I50V	9
de Faria [39]	2008	Brazil	Mixed	Children	Atopic	88	202	I50V	6
Liu [40]	2008	China	Asian	Adults	NA	108	88	Q551R	5
Trajkov [41]	2008	Macedonia	Caucasian	Adults	NA	74	249	Q551R	8
Sun [42]	2008	China	Asian	Adults	NA	82	50	Q551R	6
Amirzargar [43]	2009	Iran	Caucasian	Children	NA	59	139	Q551R	8
Llanes [44]	2009	Spain	Caucasian	Adults	Atopic	109	50	I50V, Q551R	8
Wang [45]	2009	China	Asian	Children	NA	449	512	I50V, Q551R	10
Xu [46]	2009	China	Asian	Children	NA	128	82	I50V, Q551R	7
Beghe [47]	2010	UK Italy	Caucasian	Adults	Mixed	299	176	I50V, Q551R	9
Berce [48]	2010	Slovenia	Caucasian	Children	Mixed*	106	89	Q551R	8
Bottema [49]	2010	Netherlands	Caucasian	Adults	Atopic	118	102	I50V, Q551R	9
Michel [50]	2010	German	Caucasian	Children	NA	703	658	I50V	11
Undarmaa 1 [51]	2010	Japan	Asian	Children	Atopic	325	336	I50V, Q551R	9
Undarmaa 2 [51]	2010	Japan	Asian	Adults	Atopic	367	676	I50V, Q551R	9
Wu [52]	2010	China	Asian	Children	NA	252	227	I50V, Q551R	8
Fan [53]	2010	China	Asian	Adults	NA	62	30	Q551R	5
GeMDBJ [54]	2010	Japan	Asian	NA	NA	770	2375	Q551R	NA
Murk [55]	2011	USA	Mixed*	Children	Atopic	99	480	I50V, Q551R	7
Su [56]	2012	China	Asian	Children	NA	235	1075	I50V	10

* Different data could be separately extracted.

NA, not available.

**Table 2:** Distribution of *IL4RA* I50V and Q551R polymorphisms among patients and controls.

Studies	Asthma			Control			HWE (*P* value)
*IL4RA* I50V	II	IV	VV	II	IV	VV	
Mitsuyasu	142	125	93	20	57	43	0.880
Noguchi	10	57	34	16	44	41	0.470
Takabayashi	17	49	34	16	36	48	0.048
Howard	30	53	26	25	42	20	0.770
Leung	23	38	15	19	35	16	0.988
Mújica-López	15	13	2	19	11	2	0.811
Risma	67	98	35	20	32	13	0.975
Beghe	46	101	39	199	340	131	0.509
Hytonen	28	49	23	29	51	20	0.777
Lee	54	133	69	20	51	29	0.777
Yang	6	21	7	8	16	5	0.534
Bernstein	24	29	9	19	43	13	0.182
Kabesch	25	32	16	256	375	142	0.820
Melen	169	256	96	148	253	108	0.995
Deng	24	47	29	9	33	58	0.189
Tang	33	36	34	18	23	21	0.044
Battle	76	131	55	56	86	32	0.919
López	29	42	17	34	47	15	0.852
Zhang W	84	154	65	99	180	76	0.729
Zhang H	78	168	106	17	53	44	0.873
Chan	79	159	57	49	80	38	0.626
de Faria	20	52	16	67	96	39	0.661
Llanes	35	52	22	16	23	11	0.617
Wang	139	201	105	136	250	124	0.667
Xu	50	54	24	18	43	21	0.649
Beghe	84	149	66	51	88	37	0.933
Bottema	34	59	25	30	51	21	0.937
Michel	162	351	190	122	322	214	0.964
Undarmaa 1	133	150	42	127	159	50	0.984
Undarmaa 2	138	174	55	238	326	112	0.984
Wu	46	131	75	59	110	58	0.642
Murk	28	49	23	142	236	106	0.670
Su	80	101	54	280	505	290	0.048
*IL4RA* Q551R	RR	QR	QQ	RR	QR	QQ	
Mitsuyasu	7	76	217	1	21	78	0.751
Rosa-Rosa	19	49	81	1	20	36	0.339
Heinzmann	16	80	104	5	34	61	0.926
Sandford	7	69	145	5	43	95	0.961
Takabayashi	2	27	71	5	16	79	0.003
Hakonarson	3	28	63	4	37	57	0.505
Howard	8	39	104	5	36	73	0.834
Risma	19	84	97	3	20	42	0.765
Beghe	11	62	114	36	229	404	0.635
Cui	23	89	129	4	41	130	0.720
Hytonen	6	35	59	3	30	67	0.871
Lee	9	58	189	5	11	84	0.000
Isidoro-García	1	41	91	4	26	49	0.820
Hu	19	66	90	4	41	130	0.720
Sun	4	19	59	3	10	46	0.033
Bernstein	4	17	40	2	30	43	0.222
Melen	28	184	309	25	174	310	0.927
Gui	2	15	33	2	14	34	0.716
López	8	39	49	6	42	38	0.215
Mak	4	81	200	9	91	191	0.642
Zhang W	19	87	197	22	93	240	0.003
Zhang H	8	87	257	0	27	87	0.152
Liu	15	79	54	0	78	10	0.000
Trajkov	3	27	44	11	78	212	0.262
Sun	2	15	65	0	8	42	0.539
Amirzargar	1	25	32	2	30	106	0.941
Llanes	3	37	69	2	14	34	0.716
Wang	9	112	326	12	140	360	0.710
Xu	8	49	71	2	29	51	0.364
Beghe	14	103	182	8	58	110	0.920
Berce	6	28	72	6	27	56	0.285
Bottema	7	43	68	7	41	54	0.835
Undarmaa 1	7	73	245	8	86	242	0.913
Undarmaa 2	12	102	253	10	154	512	0.681
Wu	8	61	183	4	55	168	0.837
Fan	6	8	48	3	2	25	0.000
GeMDBJ	20	194	556	46	588	1741	0.655
Murk	16	38	45	31	186	263	0.806

HWE, Hardy-Weinberg equilibrium.

### Quantitative data synthesis

#### *IL4RA* I50V polymorphism

**Figure 2:**
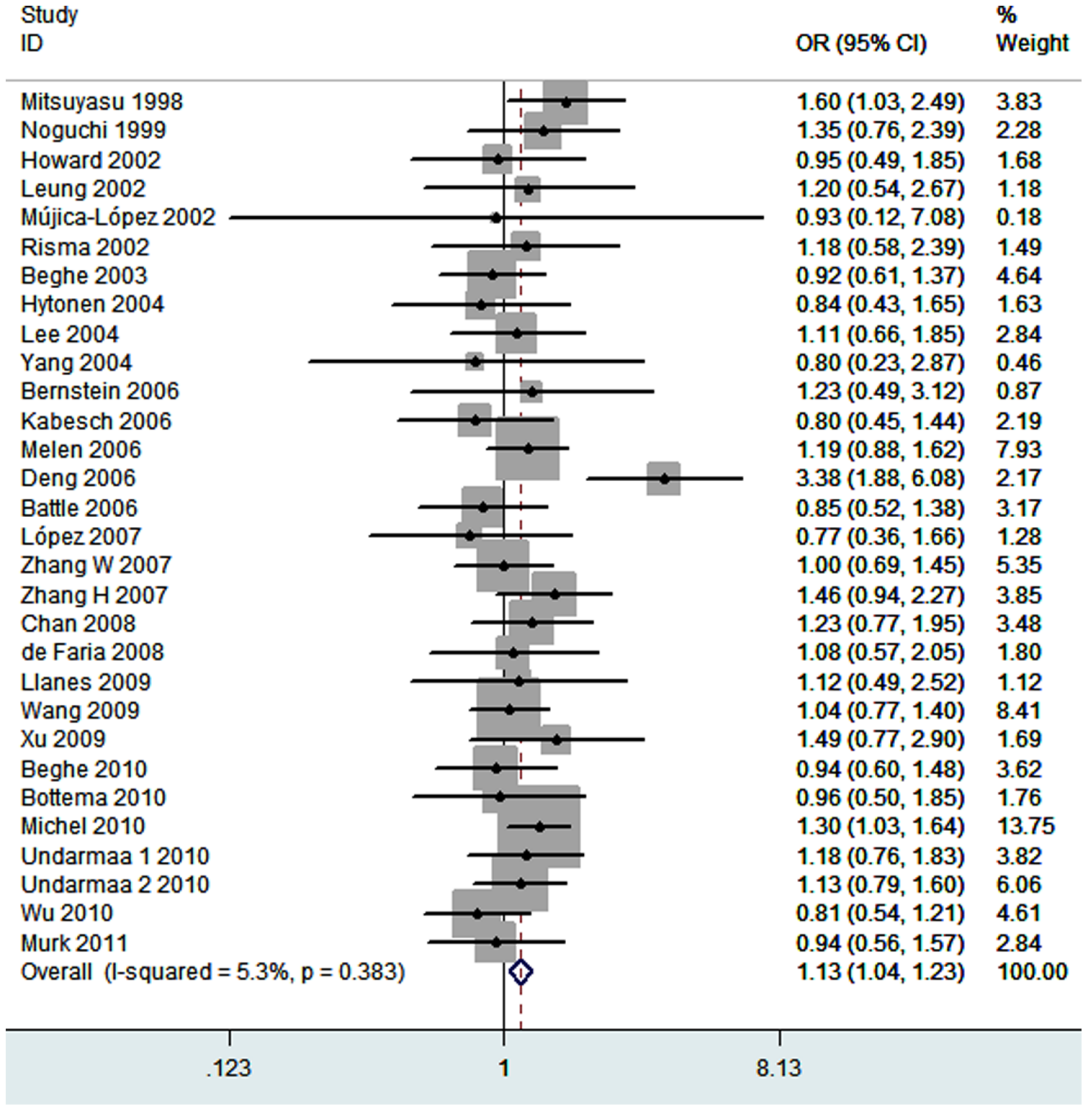
Meta-analysis for the association between asthma risk and the *IL4RA* I50V polymorphism.

Thirty studies investigated the association between the I50V polymorphism and asthma risk. The total sample sizes for case and control groups were 6442 and 7240, respectively. The estimated OR1, OR2 and OR3 values were 1.14 (*P* = 0.08), 1.09 (*P* = 0.06), and 1.06 (*P* = 0.35), respectively (**Table** **3**). These estimates suggested a dominant genetic model; therefore II and IV were combined and compared with VV. The pooled OR was 1.13 (95% CI 1.04–1.23, *P* = 0.005) (**Figure** **2**). There was no significant heterogeneity (*I^2^* = 5%, *P* = 0.38). In the stratified analysis by ethnicity, no significant association was found for the studies in Asians (OR = 1.23, 95% CI 1.05–1.45, *P* = 0.01) or Caucasians (OR = 1.10, 95% CI 0.96–1.26, *P* = 0.15). In the subgroup analysis by age, the *IL4RA* I50V polymorphism was not associated with pediatric asthma risk (OR = 1.15, 95% CI 1.03–1.29, *P* = 0.01) or adult asthma risk (OR = 1.08, 95% CI 0.91–1.27, *P* = 0.39). In the subgroup analysis according to atopic status, the *IL4RA* I50 V polymorphism was not significantly associated with the risk of atopic asthma (OR = 1.19, 95% CI 1.01–1.40, *P* = 0.04) or non-atopic asthma risk (OR = 0.92, 95% CI 0.63–1.35, *P* = 0.67).

**Table 3:** Determination of the genetic effects of *IL4RA* polymorphisms on asthma and subgroup analysis.

		Sample size		No. of	Test of association				Heterogeneity		
Polymorphisms	Study	case	control	studies	OR (95 % CI)	*Z*	*P *Value	Model	*χ* ^2^	*P *Value	*I* ^2^ (%)
*IL4RA* I50V											
II vs. VV	Overall	3314	3707	29	1.14 (0.98–1.33)	1.76	0.08	R	51.82	0.006	44.0
IV vs. VV	Overall	4564	5172	29	1.09 (1.00–1.20)	1.90	0.06	F	18.52	0.93	0.0
II vs. IV	Overall	5006	5601	29	1.06 (0.94–1.19)	0.94	0.35	R	45.83	0.02	37.0
II+IV vs. VV	Overall	6442	7240	29	1.13 (1.04–1.23)	2.78	0.005	F	30.63	0.38	5.0
II+IV vs. VV	Asian	3394	2987	14	1.23 (1.05–1.45)	2.51	0.01	R	21.06	0.07	38.0
II+IV vs. VV	Caucasian	2480	3265	11	1.10 (0.96–1.27)	1.44	0.15	F	5.71	0.84	0.0
II+IV vs. VV	Children	3500	4366	15	1.15 (1.03–1.29)	2.46	0.01	F	16.56	0.28	15.0
II+IV vs. VV	Adult	1821	1835	11	1.08 (0.91–1.27)	0.86	0.39	F	5.13	0.88	0.0
II+IV vs. VV	Atopic	1919	2368	12	1.19 (1.01–1.40)	2.10	0.04	F	9.13	0.61	0.0
II+IV vs. VV	Non-atopic	235	285	3	0.92 (0.63–1.35)	0.42	0.67	F	0.78	0.68	0.0
*IL4RA* Q551R											
RR vs. QQ	Overall	4702	6144	32	1.46 (1.15–1.87)	3.05	0.002	R	44.54	0.05	30.0
QR vs. QQ	Overall	6441	8326	32	1.11 (1.00–1.24)	1.92	0.05	R	53.54	0.007	42.0
RR vs. QR	Overall	2357	2718	32	1.35 (1.12–1.63)	3.11	0.002	F	24.09	0.81	0.0
RR vs. QR+QQ	Overall	6750	8594	32	1.46 (1.22–1.75)	4.14	<0.0001	F	37.07	0.21	16.0
RR vs. QR+QQ	Asian	3974	5263	14	1.72 (1.31–2.25)	3.91	<0.0001	F	17.54	0.18	26.0
RR vs. QR+QQ	Caucasian	2642	3185	17	1.09 (0.86–1.38)	0.71	0.48	F	11.96	0.75	0.0
RR vs. QR+QQ	Children	2357	2784	12	1.50 (1.13–1.99)	2.78	0.005	F	14.21	0.22	23.0
RR vs. QR+QQ	Adult	2749	2479	16	1.36 (1.00–1.84)	1.98	0.05	F	16.32	0.36	8.0
RR vs. QR+QQ	Atopic	2533	2868	16	1.88 (1.27–2.79)	3.15	0.002	R	22.95	0.09	35.0
RR vs. QR+QQ	Non-atopic	445	747	7	1.77 (0.97–3.23)	1.85	0.06	F	6.41	0.38	6.0

Bonferroni correction was applied (*P*<0.00714). vs., versus; R, random-effects model; F, fixed-effects model; HWE, Hardy-Weinberg equilibrium.

Cumulative meta-analyses were conducted. A tendency toward significant association with asthma risk was found (**Figure** **S1**). We performed a sensitivity analysis to evaluate the stability of the meta-analysis. As shown in **Figure** **S2**, the statistical significance of the result was not altered when any single study was omitted. The funnel plot did not reveal evidence of obvious asymmetry (**Figure** **S3**). The result was further supported by Egger's test (*P* = 0.601).

#### *IL4RA* Q551R polymorphism

**Figure 3:**
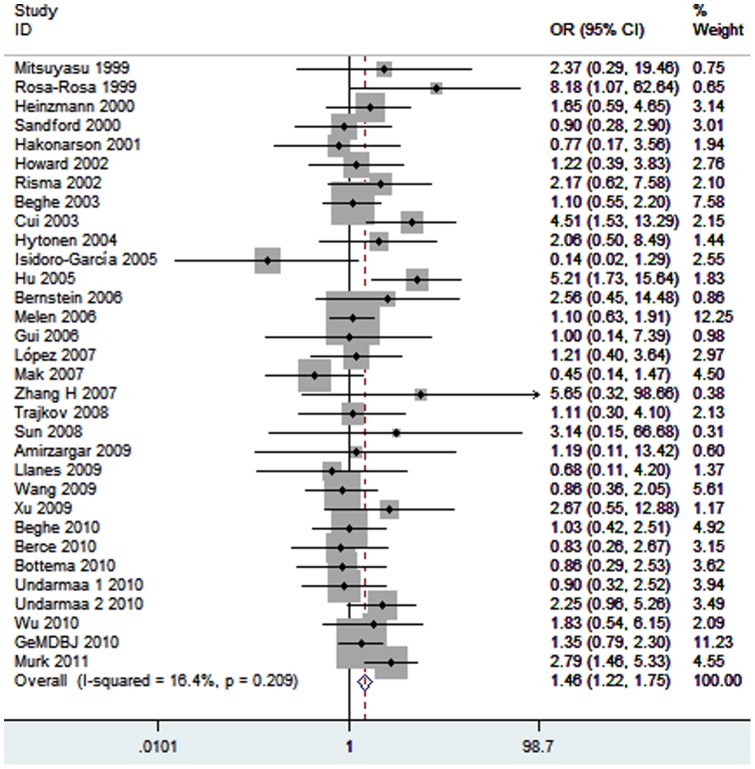
Meta-analysis for the association between asthma risk and the *IL4RA* R551Q polymorphism.

Thirty-two studies identified an association between the *IL4RA* Q551R polymorphism and asthma risk. A total of 6750 cases and 8594 controls were included in this meta-analysis. The estimated OR1, OR2 and OR3 values were 1.46 (*P* = 0.002), 1.11 (*P* = 0.05), and 1.35 (*P* = 0.002), respectively (**Table** **3**). Thus, these estimates suggested a recessive genetic model; therefore QR and QQ were combined and compared with RR. The pooled OR was 1.46 (95% CI 1.22–1.75, *P*<0.0001) (**Figure** **3**). No significant heterogeneity was observed (*I^2^* = 16%, *P* = 0.21). Subgroup analysis was performed by ethnicity. Statistically significant findings were found in Asians (OR = 1.72, 95% CI 1.31–2.25, *P*<0.0001) but not in Caucasians (OR = 1.09, 95% CI 0.86–1.38, *P* = 0.48). In the stratified analysis by age group, a statistically significantly increased asthma risk was found among children (OR = 1.50, 95% CI 1.13–1.99, *P* = 0.005), but no significant risk was found among adult asthmatic patients (OR = 1.36, 95% CI 1.00–1.84, *P* = 0.05). In terms of atopic status, we found a significant association between this polymorphism and atopic asthma risk (OR = 1.88, 95% CI 1.27–2.79, *P* = 0.002). However, there was no significant association with non-atopic asthma (OR = 1.90, 95% CI 0.94–3.84, *P* = 0.07).

Evidence from a cumulative meta-analysis showed that the results were consistent over time (**Figure** **S4**). A sensitivity analysis showed no substantial modification of the estimates after exclusion of individual studies (**Figure** **S5**). The shape of the funnel plot was symmetrical (**Figure** **S6**). Egger's test indicated the absence of publication bias (*P* = 0.773).

## Discussion

On the basis of 50 eligible case-control studies, this meta-analysis comprehensively evaluated the association between the *IL4RA* I50V and Q551R polymorphisms and asthma risk. In terms of the *IL4RA* I50 V polymorphism, we found that individuals with the 50I allele (II or IV) showed an increased risk of asthma in the overall population. However, in the subgroup analyses based on ethnicity, age group, and atopic status, no significant associations were observed after Bonferroni correction. A significant association was also noted for the *IL4RA* Q551R polymorphism. This result suggests that individuals carrying the RR genotype had an increased asthma risk. There is no significant difference in the frequencies of *IL4RA* Q551R alleles between Asians and Caucasians with asthma (http://asia.ensembl.org); however, analysis stratified by ethnicity showed a significant association with asthma in Asians, but not in Caucasians. It is possible that different lifestyles, diets, and environments may account for this apparent discrepancy. These issues should be investigated in future studies. In the subgroup analysis stratified by age group, the *IL4RA* Q551R polymorphism was associated with increased pediatric asthma risk. These results demonstrate that even the same variant in the same gene may have a different effect on the pathogenesis and occurrence of asthma in different individuals. To the best of our knowledge, no previous study has assessed the age-specific influence of *IL4RA* Q551R on asthma risk, and further studies are needed to address the effect of this polymorphism on asthma risk in different age groups. We also carried out a subgroup analysis according to atopic status. There was a significant association between this polymorphism and atopic asthma risk, suggesting that the *IL4RA* Q551R polymorphism may play a role in the etiology of atopic asthma. IgE-mediated immune responses are best known for their involvement in allergies. Cornejo-García et al. [65] showed that the *IL4RA* Q551R polymorphism was associated with IgE against prevalent allergens and with total IgE. The *IL4RA* Q551R polymorphism may therefore be a relevant marker for allergies and atopic asthma development.

IL-4Rα has been shown to play a pivotal role in the pathogenesis of Th2 inflammation and asthma. For example, Kelly-Welch et al. [66] reported that IL-4Rα-deficient mice engrafted with bone marrow derived from IL-4Rα-expressing mice failed to develop goblet-cell metaplasia in response to allergic airway inflammation. In addition, deletion of the gene encoding IL-4Rα rendered mice resistant to the induction of experimental allergic asthma [67]. Mitsuyasu et al. [7] documented that the IL-4Rα 50I variant significantly upregulated the receptor response to IL-4, with resultant increased activation of STAT6, and hence increased cell proliferation and increased IgE production. Furthermore, Rosenwasser et al. [68] showed that peripheral blood mononuclear cells derived from individuals carrying the 551R variant had enhanced IL-4 responsiveness compared with 551Q. It is therefore possible that these two polymorphisms could influence the susceptibility to asthma. The 50I and 551Q variants may be associated with increased asthma risk. The results of this meta-analysis strongly support this hypothesis.

A previous meta-analysis by Loza and Chang has focused on the relationship between these polymorphisms and asthma risk [57], and concluded that the I50 V polymorphism was not significant associated with asthma. However, only six studies of the I50 V polymorphism were included in that meta-analysis. A positive association between this polymorphism and asthma could therefore not be ruled out, because studies with small sample sizes may have had insufficient statistical power to detect any slight effect. Our current meta-analysis included 30 studies (6442 cases and 7240 controls), and found a moderate but significant association. Furthermore, this meta-analysis addressed the methodological issues such as cumulative meta-analysis and sensitivity analysis.

Results from our meta-analysis were stable and reliable. First, sensitivity analyses and cumulative meta-analyses revealed that the results were robust. Second, there was no significant heterogeneity in most of the comparisons. Third, funnel plots and Egger's tests found no significant publication bias. However, some limitations should be addressed. First, the numbers of published studies involving African Americans and Mexicans were limited. Second, the overall outcome was based on unadjusted data, whereas a baseline risk-adjusted analysis could be performed if individual data were available to allow adjustment. Third, asthma is a complex disease with multifactorial etiology. A lack of original data from the eligible studies limited evaluation of the effects of the gene-gene and gene-environment interactions during asthma development. These gene-environment and gene-gene interactions should be further evaluated. Fourth, even though no significant publication bias was found by funnel plot analysis and formal statistical tests, it was impossible to exclude potential publication bias completely, because small studies with null results tend not to be published. Finally, all the studies included in this meta-analysis used a case-control design, which was susceptible to recall and selection biases. In addition, there was a risk of residual confounding by unmeasured factors.

In conclusion, this meta-analysis found significant associations between the *IL4RA* I50V and Q551R polymorphisms and asthma risk. Further studies in more ethnic groups, especially African Americans and Mexicans, are warranted to validate these results.

## Supporting Information

Figure S1 **Cumulative meta-analysis of associations between the** ***IL4RA*** **I50V polymorphism and asthma risk.**
(TIF)

Figure S2 **Sensitivity analysis for the** ***IL4RA*** **I50V polymorphism with asthma risk.**
(TIFF)

Figure S3 **Funnel plot for asthma risk and the** ***IL4RA*** **I50V polymorphism.**
(TIFF)

Figure S4 **Cumulative meta-analysis of associations between the** ***IL4RA*** **R551Q polymorphism and asthma risk.**
(TIF)

Figure S5 **Sensitivity analysis for the** ***IL4RA*** **R551Q polymorphism with asthma risk.**
(TIFF)

Figure S6 **Funnel plot for asthma risk and the** ***IL4RA*** **R551Q polymorphism.**
(TIFF)

Table S1 **Scale for quality assessment of molecular association studies of asthma.**
(DOCX)

Checklist S1 **Checklist of items to include when reporting a systematic review or meta-analysis.**
(DOC)
