# Supplementary figures and images for: Association between Interleukin-4 Receptor α Chain (IL4RA) I50V and Q551R Polymorphisms and Asthma Risk: An Update Meta-Analysis
Source: PLoS One. 2013 Jul 26;8(7):e69120. doi: 10.1371/journal.pone.0069120 (PMC3724857; doi:10.1371/journal.pone.0069120)

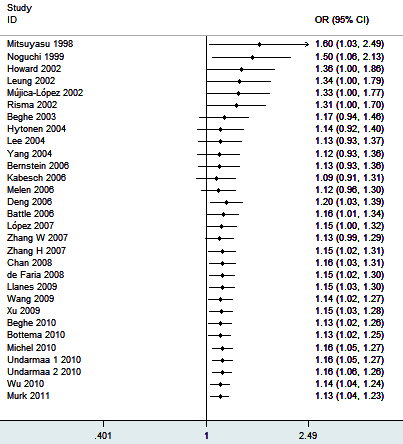

Supplement: Figure S1 — Cumulative meta-analysis of associations between the IL4RA I50V polymorphism and asthma risk. (TIF) [file pone.0069120.s001.tif]

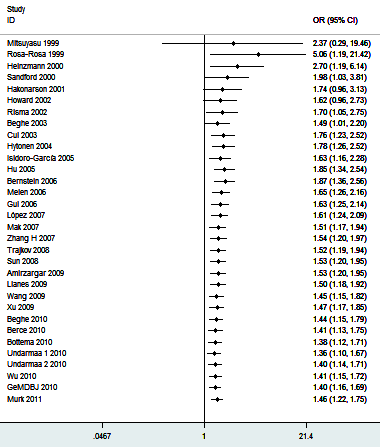

Supplement: Figure S4 — Cumulative meta-analysis of associations between the IL4RA R551Q polymorphism and asthma risk. (TIF) [file pone.0069120.s004.tif]
