# Supplementary material for: Association between Interleukin-4 Receptor α Chain (IL4RA) I50V and Q551R Polymorphisms and Asthma Risk: An Update Meta-Analysis
Source: PLoS One. 2013 Jul 26;8(7):e69120. doi: 10.1371/journal.pone.0069120 (PMC3724857; doi:10.1371/journal.pone.0069120)
Supplement: Table S1 — Scale for quality assessment of molecular association studies of asthma. (DOCX) [file pone.0069120.s007.docx]

Table S1. Scale for quality assessment of molecular association studies of asthma

| Criteria | Score |
| --- | --- |
| Representativeness of cases |  |
| Consecutive/randomly selected from case | 2 |
| population with clearly defined sampling |  |
| frame |  |
| Consecutive/randomly selected from case | 1 |
| population without clearly defined sampling |  |
| frame or with extensive inclusion/exclusion  criteria |  |
| No method of selection described | 0 |
| Representativeness of controls |  |
| Controls were consecutive/randomly drawn | 2 |
| from the same sampling frame |  |
| (ward/community) as cases |  |
| Controls were consecutive/randomly drawn | 1 |
| from a different sampling frame as cases |  |
| Not described | 0 |
| Ascertainment of asthma |  |
| Clearly described objective criteria for | 2 |
| diagnosis of asthma |  |
| Diagnosis of asthma by patient self-report or | 1 |
| by patient history |  |
| Not described | 0 |
| Ascertainment of controls |  |
| Controls were tested to screen out asthma | 2 |
| Controls were subjects who did not report | 1 |
| asthma |  |
| Not described | 0 |
| Genotyping examination |  |
| Genotyping done under blinded condition | 1 |
| Unblinded or not mentioned | 0 |
| Hardy-Weinberg equilibrium |  |
| Hardy-Weinberg equilibrium in control group | 2 |
| Hardy-Weinberg disequilibrium in control | 1 |
| group |  |
| No checking for Hardy-Weinberg equilibrium | 0 |
| Association assessment |  |
| Assess association between genotypes and | 2 |
| asthma with appropriate statistics and |  |
| adjustment for confounders |  |
| Assess association between genotypes and | 1 |
| asthma with appropriate statistics without |  |
| adjustment for confounders |  |
| Inappropriate statistics used | 0 |
